# Myristic Acid Coated Protein Immobilised Mesoporous Silica Particles as pH Induced Oral Delivery System for the Delivery of Biomolecules

**DOI:** 10.3390/ph12040153

**Published:** 2019-10-12

**Authors:** Vivek Trivedi, Ruchir Bhomia, John C Mitchell

**Affiliations:** 1 Medway School of Pharmacy, University of Kent, Central Avenue, Chatham Maritime, Kent ME4 4TB, UK; 2 Faculty of Engineering and Science, University of Greenwich, Central Avenue, Chatham Maritime, Kent ME4 4TB, UK; bhomia.r@pg.com (R.B.); J.C.Mitchell@gre.ac.uk (J.C.M.)

**Keywords:** biomolecules, oral delivery, supercritical CO_2_, myristic acid, haemoglobin

## Abstract

Solid core drug delivery systems (SCDDS) were prepared for the oral delivery of biomolecules using mesoporous silica as core, bovine haemoglobin (bHb) as model drug and supercritical fluid (SCF) processing as encapsulation technique. The use of organic solvents or harsh processing conditions in the development of drug delivery systems for biomolecules can be detrimental for the structural integrity of the molecule. Hence, the coating on protein-immobilised particles was performed via supercritical carbon dioxide (scCO_2_) processing at a temperature lower than the melting point of myristic acid (MA) to avoid any thermal degradation of bHb. The SCDDS were prepared by bHb immobilisation on mesoporous silica followed by myristic acid (MA) coating at 43 °C and 100 bar in scCO_2_. bHb-immobilised silica particles were also coated via solvent evaporation (SE) to compare the protein release with scCO_2_ processed formulations. In both cases, MA coating provided required enteric protection and restricted the bHb release for the first two hours in simulated gastric fluid (SGF). The protein release was immediate upon the change of media to simulated intestinal fluid (SIF), reaching 70% within three hours. The release from SCF processed samples was slower than SE formulations, indicating superior surface coverage of MA on particles in comparison to the SE method. Most importantly, the protein conformation remained unchanged after the release from SCDDS as confirmed by circular dichroism. This study clearly demonstrates that the approach involving protein immobilisation on silica and scCO_2_ assisted melt-coating method can protect biomolecules from gastric environment and provide the required release of a biologic in intestine without any untoward effects on protein conformation during processing or after release.

## 1. Introduction 

Parenteral administration is the most common route for therapeutic peptide/protein delivery [1]. However, the short half-life of biologics due to their sensitive nature and chronic therapy requirements in majority of treatments make repetitive dosing unavoidable. Injectables, the most common route of delivery, also require formulation improvements. Current developments in invasive delivery of biomolecules include formulations based on encapsulation of protein into lipid based microparticles and a polymeric matrix to control the release and achieve a prolonged effect [2]. Since the matrix remains in the body for a considerable period, materials used to formulate them must have non-toxic degradation products. Polyesters, polyanhydrides, and naturally occurring materials, including gelatin, alginate and chitosan, are amongst the most commonly used polymers to develop sustained release protein formulations [3]. However, most of these still fail to answer issues related to cost, frequent injections and low patient compliance. Hence, finding alternatives to parenteral delivery is highly important as the application of biologics as therapeutics increases. Therefore, research in non-invasive administration of biomolecules is required. The development of drug delivery systems for biologics is a complex process and cannot be compared to small molecules due to the size and sensitivity of these molecules [4]. There are very few peptides/proteins that are administered through a route other than parenteral. Examples of marketed non-parenteral protein formulations include Fortical^®^ (nasal calcitonin spray) for the treatment of post-menopausal osteoporosis and Oralyn^®^ (buccal insulin formulation) for terminally ill patients. Alternatives to parenteral delivery are challenging due to issues related to other potential routes. For example, protein denaturation due to stomach acid, enzymatic degradation, mucocilliary clearance and impermeability across the intestinal wall are well known issues associated with oral delivery. Another major challenge in developing novel drug delivery systems for biologics is linked to exposure of biomolecules to harsh processing conditions such as high shear stress, temperature and organic-water solvent interface, which may lead to structural changes in proteins [5]. For example, encapsulation can be achieved by a wide range of processes, such as spray-drying, solvent evaporation, extrusion, coacervation, etc. However, these processes require the use of organic solvents and subject the drug to high stress and/or temperature. Therefore, processing materials that are sensitive to these conditions, e.g., proteins and peptides, is challenging via conventional techniques.

Supercritical fluid technology (SCFT) can provide a solution to the problems associated with conventional encapsulation methods. The advantages of SCFT include avoiding the use of organic solvent, operation at low temperatures, and the ability to work in oxygen- and water-free environment to prevent thermal, oxidative and solvent-induced degradation of a material [6]. scCO_2_ is the most commonly employed solvent for the particle engineering and encapsulation purposes as it allows processing at comparatively low pressures (critical pressure: 72.8 bar) and temperatures (critical temperature: 31.1 °C) and it is chemically inert, easily available, environmentally benign and easy to remove [7].

Most protein drug delivery systems are based on biodegradable polymeric nanoparticles, liposomes and dendrimers [8]. A controlled release is obtained from these materials upon structural degradation of the carrier triggered by various chemical factors, such as pH and temperature. However, premature release of drugs still remains an issue with these systems. The use of inorganic material has been also investigated by many to overcome the problem of premature release and to provide a sustained delivery. Among the many stable biocompatible excipients, silica is generally the material of choice and has been studied in many drug delivery systems [9,10]. Properties such as biocompatibility, hydrophilicity and protection to internal payload make mesoporous (pore size 2–50 nm) silica particles a perfect candidate for drug delivery. Moreover, the release rate can also be controlled by tailoring the size and shape of these particles [11]. Another important advantage of silica is the ease of its surface modification to optimise the drug loading and release kinetics [12]. Silica is also considered to have better a biocompatibility in comparison with other metal oxides such as titanium and iron oxide [13]. Artificial implants of silica and its composites are known to have osteogenic properties and it is very well established that silica is able to store and gradually release drugs like antibiotics and other small molecules [9,13]. Moreover, it is also known that the biocompatibility of several drug delivery systems such as biopolymers, micelles and magnetic nanoparticles can be enhanced by the use of silica [13,14,15]. Hence, silica can be an excellent carrier for biomolecules and that is why it is used as a core material for protein immobilisation in the present study. 

Myristic acid (MA) is a saturated fatty acid with a long hydrocarbon side chain and carboxyl group. It is a generally regarded as a safe (GRAS) material and has found numerous applications in pharmaceutical and food industry as it is easily available, biologically inert, nontoxic and biocompatible [16]. Saturated long chain fatty acids are commonly used in various pharmaceutical applications and are frequently studied in the development of drug delivery systems. Examples of the fatty acids in drug delivery include implants containing insulin, ofloxacin-loaded palmitic acid solid lipid nanoparticles, stearic acid nanoparticles loaded with cyclosporine A, encapsulation of paclitaxel into lauric acid based micelles and cefuroxime-axetil containing stearic acid microparticles [17,18,19,20,21]. Similarly, MA coating of chitosan salts containing vancomycin hydrochloride provided the required enteric properties to obtain colon-targeted drug release [22]. The favourable physico–chemical characteristics of MA related to its enhanced solubility at alkaline pH due to the ionisation of the carboxylic acid group in comparison to acidic pH can be utilised in developing formulations with essential enteric properties. 

The rationale behind SCDDS is very simple and involves immobilisation of a biologic on mesoporous particles to reduce molecular flexibility and improve the stability against pH, temperature and enzymatic degradation [23,24]. The second step involves coating of MA without exposing biomolecules to harsh conditions, via scCO_2_ processing to obtain second layer of protection and the desired release in the required environment. The aim of this study was to formulate SCDDS for the oral delivery of biologics with required enteric properties to ensure safe passage of biomolecules to thr upper intestine using bHb as a model drug, silica as the core material and scCO_2_ as the media to obtain MA coating. The rationale behind this work was to find a cost-effective drug delivery system with required enteric properties for biomolecules.

## 2. Results and Discussion

### 2.1. Maximum bHb Adsorption and Adsorption Kinetics

**Figure 1:**
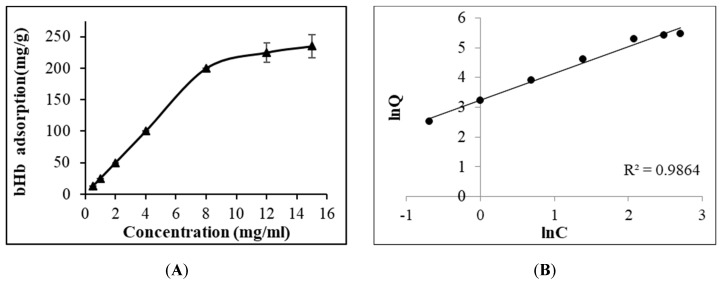
Adsorption isotherm of bHb immobilization on S_FP_ in pH 6 phosphate buffer at 25 °C (**A**) and Freundlich model fitting (**B**).

It is known that proteins contain a net positive charge below their isoelectric point (pI) and have an overall negative charge above it. The rate of protein adsorption is likely to be high when the adsorbent surface and protein bear opposite charges. This higher rate of adsorption in these conditions is primarily driven by the electrostatic attractions which accelerates the protein migration towards the oppositely charged surface [25]. The adsorption studies presented in this study were performed in pH 6 phosphate buffer (0.133 M) due to the overall positive charge on bHb, considering the pI of bHb to be 7.1 [26] and a negative charge on silica particles such that the ionic interactions could be encouraged. Another reason for the selection of this pH was the higher protein stability at pH 6 compared to pH 7 and 8 under stirring at room temperature [27,28]. The bHb adsorption on silica (Figure 1) increased linearly with the increase in protein concentration, reaching a plateau at 12 mg/mL (225 mg/g) with only a slight increase in protein immobilisation at 15 mg/mL (236 mg/g).

The data for bHb adsorption on silica particles was fitted with both Langmuir and Freundlich models, resulting in R^2^ values of 0.7915 and 0.9864, respectively. The Freundlich-type adsorption of bHb on S_FP_ indicated that it is a multi-layer process in which the amount of adsorbed solute per unit adsorbent mass increases gradually with the increase in bHb concentration [29]. 

**Figure 2:**
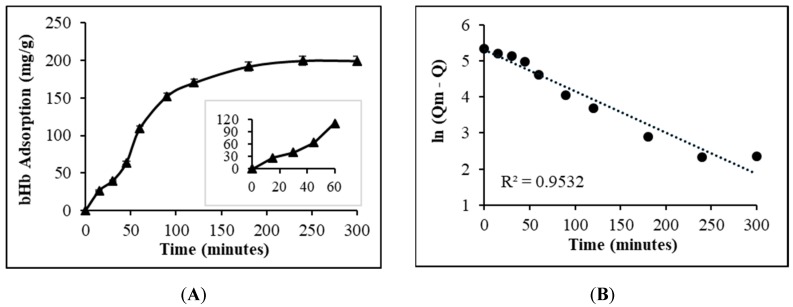
Adsorption kinetics of 8 mg/mL bHb solution on S_FP_ in pH 6 phosphate buffer at 25 °C (**A**) and Pseudo-first order kinetic fitting (**B**).

The adsorption kinetics of bHb adsorption on S_FP_ presented in Figure 2 indicate the high affinity of protein to silica particles. 

An almost linear increase (Figure 2, inset) in protein adsorption during the first 60 min confirmed the high affinity of bHb to silica at pH 6. Protein adsorption in excess of 80% at a rate of 0.58 mg/min occurred within 120 min. These studies were restricted to 4 h due to an issue with bHb stability under agitation, as reported by Bhomia et al. [27,28]. The adsorption of bHb on S_FP_ was obtained to be of pseudo first order (R^2^ = 0.9532), indicating that protein immobilization was primarily dependent on the adsorbate concentration in the media and its propensity to migrate to the adsorbent surface [30]. 

### 2.2. Effect of Protein Adsorption on Porosity and Pore Volume

**Figure 3:**
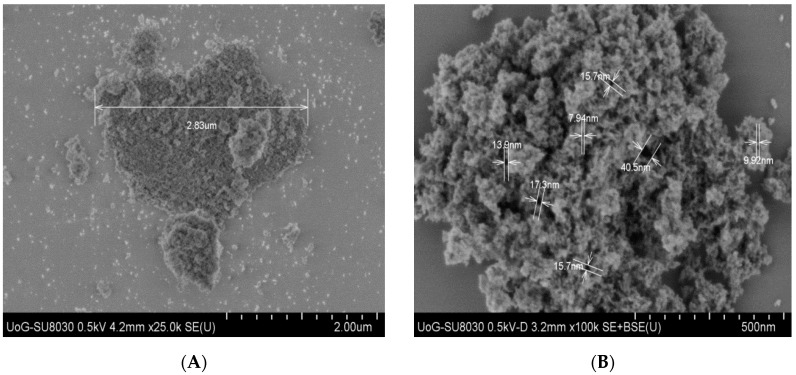
SEM micrographs of S_FP_ particle at 25 k (**A**) and 100 k (**B**) magnifications.

Syloid^®^ excipients are micronized synthetic amorphous silica gels of high purity that are widely used in pharmaceutical formulations [31,32]. The S_FP_ particles are pharmacopeia accepted and FDA-compliant nonordered mesoporous particles with an average size of 3.5 μm [33]. Micrographs (Figure 3) of S_FP_ particles confirmed that they were compacts or agglomerates of irregular shapes with the presence of a large volume of empty spaces and interconnected pores. 

**Figure 4:**
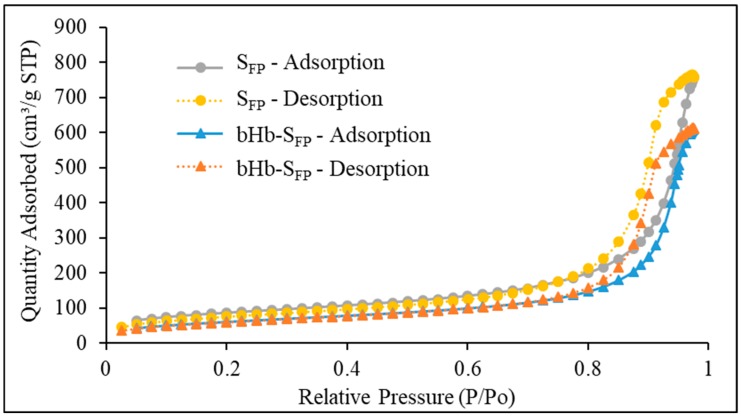
Nitrogen adsorption isotherm for S_FP_ and bHb-S_FP_.

The complete nitrogen adsorption and desorption isotherm for S_FP_ presented in Figure 4 resembled the BDDT Type IV, indicative of the mesoporous nature of the particles. The hysteresis in this isotherm resembled Type H1, meaning that these particles consisted of agglomerates or compacts of approximately uniform spheres in a regular array [34]. BET isotherm of bHb-adsorbed silica retained its Type IV nature but showed a decrease in specific surface area. The BET surface area of S_FP_ decreased from 307 m^2^/g to 226 m^2^/g after bHb adsorption. 

**Figure 5:**
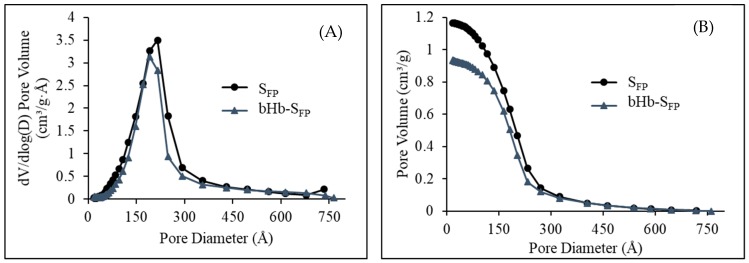
Pore size distribution (**A**) and cumulative pore volume (**B**) of S_FP_ and bHb-S_FP_.

The average pore diameter and cumulative pore volume of S_FP_ as calculated by the BJH model were 160 Å (16 nm) and 1.16 mL/g, respectively, as shown in Figure 5 [35]. A decrease in both the average pore diameter (150 Å) and the cumulative pore volume (0.9 mL/g) was observed after bHb immobilization on silica particles. 

The availability of a large available surface area after bHb adsorption may suggest that silica surface or pores within remained unoccupied by protein molecules. However, it is important to remember that the smaller pores which were easily accessible to nitrogen molecules during the BET analysis could be completely inaccessible to large molecules such as bHb. Larger available pores may potentially be able to accommodate more than one molecule within them but pores similar to or smaller than the dimensions of bHb (5.3-5.4-6.5 nm) largely remain unoccupied during the adsorption process. The decrease in the surface area and pore volume clearly indicates the presence of bHb inside the pores or the blockage of pore openings by protein molecules after the adsorption process. Similarly, an increase in the point of zero charge (PZC) for silica from pH 1.6 for bare particles to 6.7 for protein immobilized particles (closer to the IEP of bHb) also indicated coverage of particle surface with bHb molecules. 

### 2.3. SCDDS Preparation

**Figure 6:**
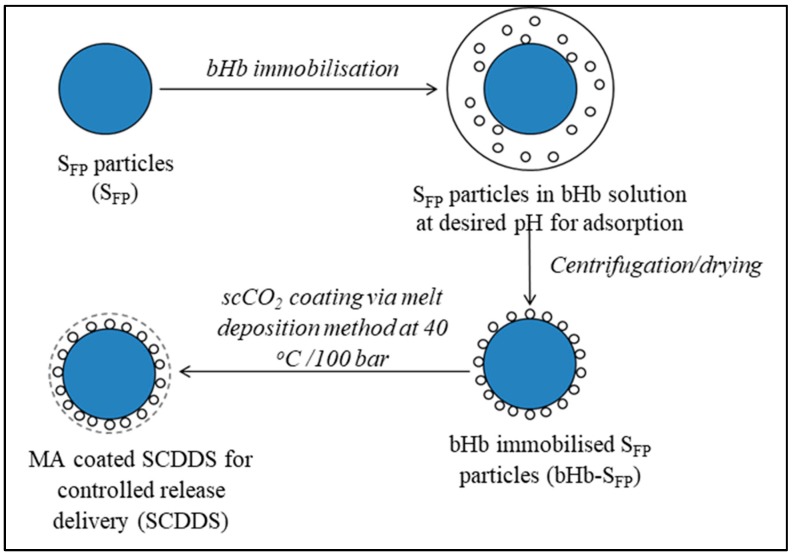
Schematic diagram of solid core drug delivery system (SCDDS) preparation.

The preparation of SCDDS involved protein immobilisation on a solid surface followed by coating to control the drug release. The protein immobilisation on a solid surface is known to reduce the molecular flexibility, leading to enhanced stability [22]. Moreover, coating thereafter ensures that these particles have optimal enteric properties to protect the protein from gastric pH and control the release [36]. The SCDDS formulation process can be summarised as follows:

As illustrated in Figure 6, the first step in this process allows the bHb molecules from the aqueous solution to be adsorbed on the silica surface which is then collected via centrifugation and dried to obtain bHb-immobilised S_FP_ particles. The protein adsorbed particles are then coated with MA via melt-deposition method in scCO_2_ to limit possible denaturation due to oxidation, hydrolysis and solvent, stress and temperature-induced degradation. The rationale behind using MA coating is also to protect the protein from gastric media due to its limited solubility in acidic conditions and to promote drug release in intestinal environment.

**Figure 7:**
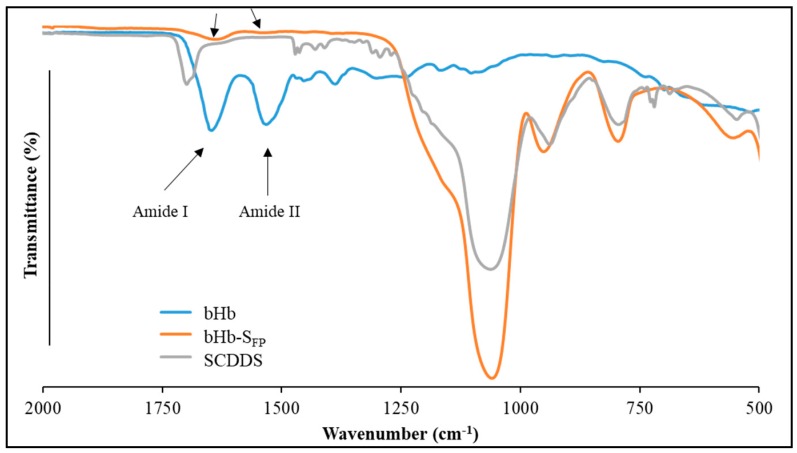
ATR-FTIR spectra of bHb, bHb-S_FP_ and SCDDS.

S_FP_, S_FP_-bHb and SCDDS were analysed using ATR-FTIR to confirm protein adsorption, coating and whether bHb went through any conformational changes due to immobilisation at the given conditions. The spectra presented in Figure 7 show amid I and amid II peaks at 1644 and 1530 cm^−1^, respectively.

Amide I and amide II bands are two major bands of the protein infrared spectrum [37]. The amide I band (between 1600 and 1700 cm^−1^) is directly related to the backbone conformation and it is mainly associated with the C=O stretching vibration. The N-H bending vibration and the C-N stretching vibration results in amide II between 1450 and 1550 cm^−1^. This amide II band is conformationally sensitive and can provide information about protein folding/unfolding. Both peaks are still present in bHb immobilised S_FP_ with reduced peak intensity but without any shift, confirming the lack of changes to protein conformation due to immobilisation. The absence of characteristic peaks (amide I and II) of protein and the emergence of the peak for carboxyl groups at 1701 cm^−1^ along with the reduction in the intensity of siloxane (Si-O-Si) band at 1055 cm^−1^ demonstrate MA coating of the silica surface. 

### 2.4. In-Vitro Release Studies at pH 1.2 and 6.8

**Figure 8:**
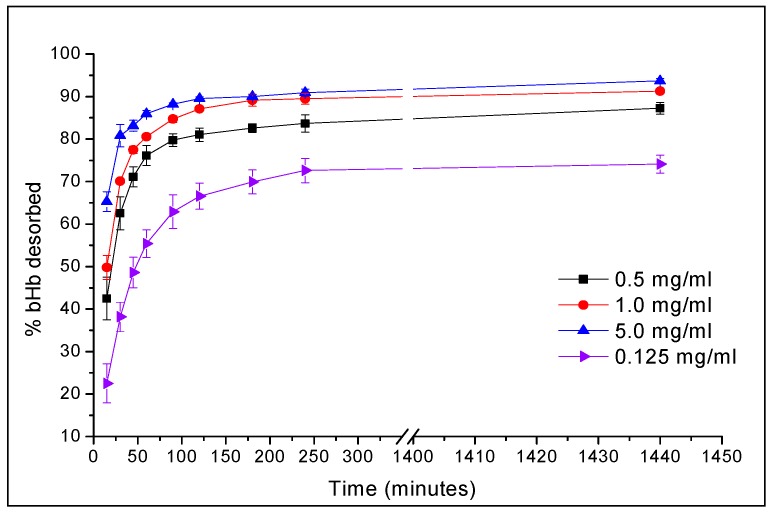
bHb desorption from S_FP_ particles in pH 6.8 phosphate buffer at 37.4 °C using F-127 as a displacer.

One of the major issues with protein immobilisation on mesoporous surfaces is the inability to obtain sufficient desorption thereafter due to the increase in the surface free energy. Hence, addition of a displacer in the dissolution media was explored to enhance the protein release from these systems. Although protein desorption can be obtained via changes in solvent ionic strength, pH and/or use of surfactants, it is important to carefully consider the choice of the displacement mechanism to ensure conformational integrity [38,39,40,41,42]. Pluronics are triblock polymers consisting of polyethylene and polypropylene blocks and act as non-ionic surfactants. Prior to release studies, experiments were performed to determine the efficiency of pluronic F127 (F127) as protein displacer and its impact on the protein conformation. Figure 8 shows the effect of F217 concentration on the protein desorption from silica surface.

**Figure 9:**
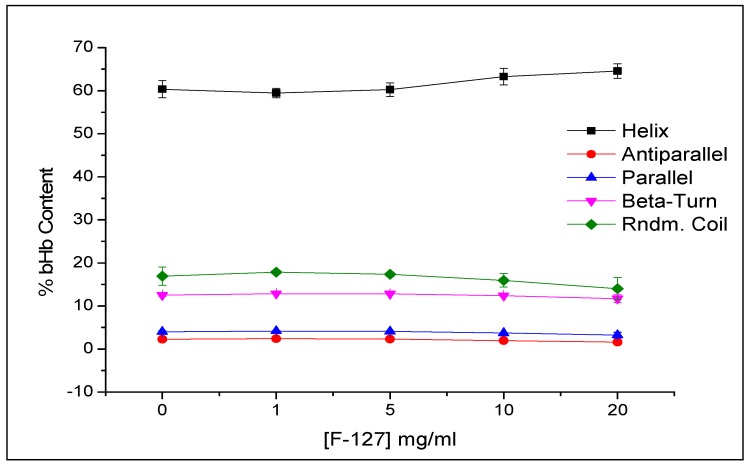
Effect of F-127 concentration on bHb secondary structures after desorption from S_FP_ in pH 6.8 phosphate buffer at 37.4 °C.

The increase in F127 concentration in the media resulted in higher protein release of up to 1 mg/mL with no real change to the release profile with the further increase of F127 to 5 mg/mL. The effect of F127 in the media on protein conformation was evaluated using CD spectroscopy, which showed no impact on the secondary structures of the bHb, as presented in Figure 9, in terms of the percentage content of five different secondary structures. These were calculated using CDNN software developed by Dr. Gerald Böhm (Institut für Biotechnologie, Martin-Luther Universität Halle-Wittenberg), which deconvolutes the CD data by cross-referencing the sample spectrum with already installed reference spectra [28].

Ionic surfactants are known to cause protein unfolding via hydrophobic and ionic interactions [43], whereas non-ionic surfactants such as pluronics have a limited effect on the protein conformation. Pluronics have also been studied as permeation enhancers which can potentially aid in the absorption of biomolecule after release [44]. In this study, PF127 was used as a displacer in the media with the aim to formulate SCDDS containing both MA and pluronic in the future to obtain a drug delivery system capable of providing sufficient release at desired conditions. Based on the findings of these experiments, bHb release from SCDDS was determined with 1 mg/mL in the dissolution media.

**Figure 10:**
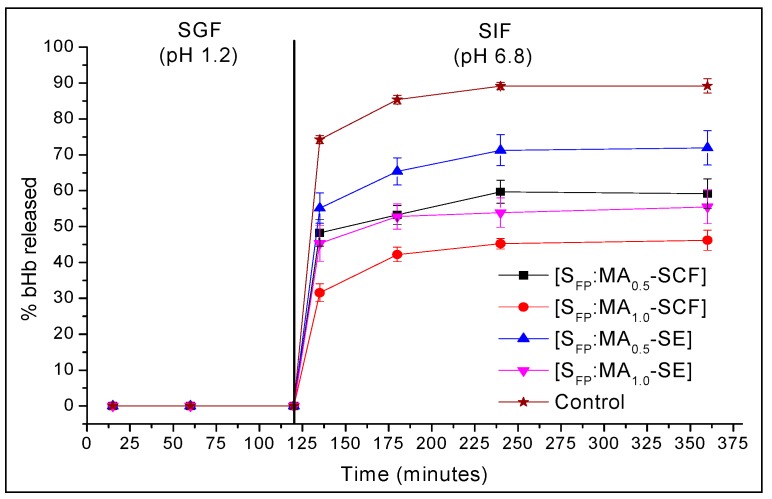
Release of bHb from MA coated S_FP_ based SCDDS prepared with both SCF and SE methods in SGF (pH 1.2) and SIF (pH 6.8) at 37.4 °C.

One of the major goals of this study was to determine whether MA coating can provide sufficient protection to a biomolecule from the harsh gastric pH. The MA coated SCDDS did not show any protein release in the first 120 min at pH 1.2 (Figure 10), suggesting that these formulations possess required enteric property to provide sufficient protection to protein from the gastric environment. 

These findings were similar to the results from Pettit et al. in which systems prepared by solvent coating of MA showed release of adsorbed protein only at alkaline pH and Cerchiara et al. also showed that FA coating imparted enteric properties to vancomycin loaded chitosan particles prepared by freeze drying [22,36]. bHb release upon exposure to SIF was immediate, which may be related to the solubility behaviour of MA. The solubility of MA increases at a higher pH, which can subsequently facilitate the continuous erosion of coating and higher protein release at the studied conditions [45]. bHb release in SIF was the highest (71%) from S_FP_:MA0.5-PEN, followed by S_FP_:MA0.5-SCF, S_FP_:MA1.0-PEN, and S_FP_:MA1.0-SCF with a release of 62%, 56% and 48%, respectively.

Although SE coating imparted the required enteric properties to the formulation, SCDDS prepared by SCF processing showed a comparatively lower and slower release in comparison to particles coated by SE. This could be due to the better coating and improved surface coverage of MA when SCF-assisted melt-coating was used in comparison to SE. The protein release was also dependent on the MA/silica ratios, where 0.5:1 showed a higher release compared to 1:1 for SCDDS prepared using both coating methods. For instance, S_FP_:MA0.5-SCF released a total of 62% bHb, which was 14% higher than the formulation containing a 1:1 (MA:silica) ratio. Similarly, the total release from SCDDS prepared by solvent evaporation was 56% (1:1) and 71% (0.5:1). The increase in FA ratio in the formulation resulted in a decreased bHb release, which is expected to have been due to the slow erosion of the MA layer above the solubility limit. Moreover, SCF processing of MA also allowed the coating to have been performed at 43 °C rather than its actual melting point of 54.5 °C, which occurred due to the dissolution of CO_2_ in a myristic acid crystalline matrix. This also ensured that bHb was not exposed to comparatively higher temperature which would be the case if melt-coating was performed at atmospheric pressure. 

The dissimilarity (F1) and similarity (F2) factors were calculated to determine whether there were any statistical differences (Table 1) between the bHb release from coated and uncoated particles. 

The F1 should be between 0 and 15, whereas F2 should be between 50 and 100 for two dissolution profiles to be considered similar [46]. For all the above formulations, F1 was higher than 15 and F2 was lower than 50. Hence, it can be concluded that the release from formulations was dissimilar to the control in every scenario.

Improper design or formulation of biologics can result in degradation, denaturation, and/or aggregation of the protein molecules, potentially causing both immunogenic side effects after administration and loss of pharmacological activity. Hence, the conformation of the released bHb was determined to understand whether the immobilisation, coating or release caused any changes to the protein’s secondary structure. 

**Figure 11:**
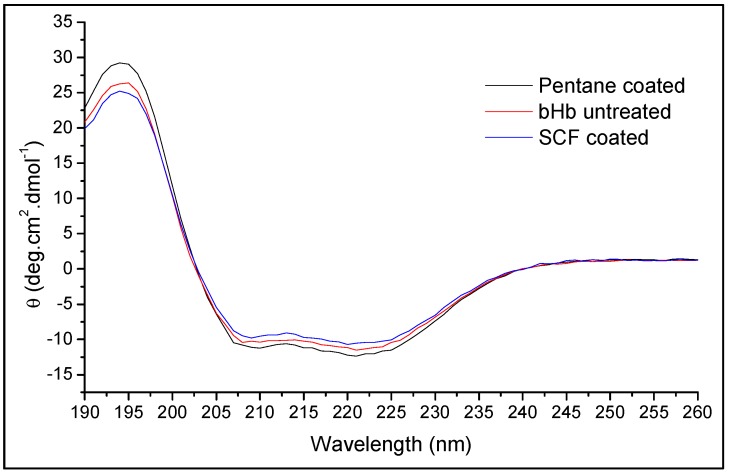
CD spectra of untreated bHb and bHb released from MA coated S_FP_ formulations.

The CD spectra of the untreated and released protein from SCDDS prepared via SCF and SE methods are presented in Figure 11. The CD spectrum of the released protein shows similar maxima and minima to untreated bHb, indicating that it largely retained its confirmation after release [47]. The obtained CD spectra were processed by the CDNN software to determine the fraction content of different secondary structures of the protein. The percentage content of secondary structures from CD spectra for protein released from MA coated formulations is shown in Table 2.

The α-helix content of the protein released from formulations was the same as the freshly prepared bHb solution, i.e., approximately 60%. Similarly, the rest of the secondary structures content was also comparable to the untreated sample, confirming the absence of any conformational changes in the bHb molecules either due to processing or exposure to the release media.

## 3. Materials and Methods

### 3.1. Materials

Bovine haemoglobin, >95% (bHb), myristic acid, ≥99% (MA), Pluronic F-127, (BioReagent grade) sodium hydrogen phosphate (≥99%), potassium dihydrogen phosphate (≥98%), hydrochloric acid (37% *v/v*), sodium hydroxide (≥97%) and pentane (98%) were purchased from Sigma Aldrich (Gillingham, UK). Syloid 244 FP silica particles (S_FP_) were kindly donated from Grace Davison (Lokeren, Belgium). Liquid CO_2_ with the purity of 99.9% was supplied by BOC ltd (Rochester, UK). All chemicals were used as received without any further purification.

### 3.2. bHb Adsorption and Kinetics 

The maximum protein immobilisation on silica particles was determined using 0.5, 1, 2, 4, 8, 12, 15 mg/mL protein solutions in pH 6 phosphate buffer. The adsorption study was performed using 400 mg of silica suspended in 10 mL solution of each concentration. The contents were stirred at 250 rpm at room temperature and the supernatant was collected, filtered using a 0.45-µm syringe filter and analysed by UV spectrophotometer (UV-2550, Shimadzu, Kyoto, Japan) after 4 h. The kinetic measurements were conducted using 8 mg/mL protein solution in pH 6 phosphate buffer. Similar to maximum bHb adsorption study, 400 mg of silica was suspended in 10 mL of protein solution. The contents were stirred at 250 rpm and UV measurements were taken after 15, 30, 45, 60, 90, 120, 180, 240 and 300 min at 285 nm. The quantity of bHb adsorbed was calculated using an indirect method, i.e., by subtracting the remaining amount of bHb in the supernatant from the initial content. The experiments were conducted in triplicate and at ambient conditions.

### 3.3. Coating of Silica Particles by SCF Processing

**Figure 12:**
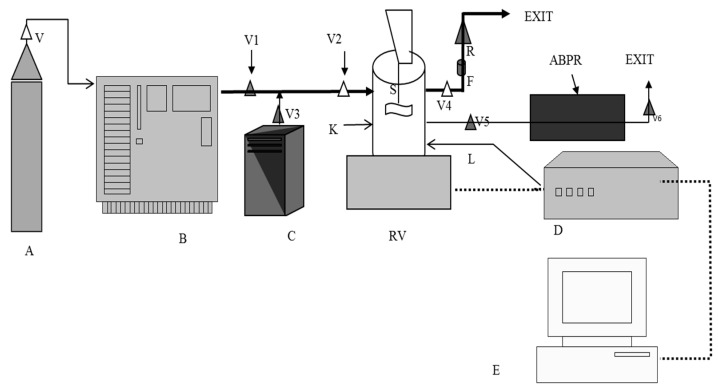
Schematic presentation of supercritical fluid (SCF) instrument [CO_2_ cylinder (**A**), chiller (**B**), CO_2_ pump (**C**), high pressure vessel (RV), automatic back pressure regulator (ABPR), controller (**D**) and display unit (**E**)].

The protein-adsorbed particles were separated from the media via centrifugation at 3700 rpm (Centrifuge 5430, Eppendorf, Stevenage, UK) for 10 min and freeze-dried at −55 °C under deep vacuum using a ScanVac CoolSafe freeze dryer (LaboGene ApS, Lillerød, Denmark). The dried particles were coated by SCF processing with MA at a temperature and pressure reported elsewhere (43 °C and 100 bar) [27,48]. The instrumental set up is detailed in Figure 12, which consists of a temperature-controlled 150 mL and 3.5-cm-thick stainless steel cylinder with sapphire windows to monitor the experiments. 

The interior of the vessel was illuminated by a light source (L) which allowed the process to be monitored live using a camera (K) directly attached to the vessel via sapphire window. An ABPR was used to control the pressure inside the vessel and to obtain controlled depressurisation. The desired pressure and temperature values were assigned and monitored from the control unit (D). The rate of CO_2_ addition and evacuation along with the live feed from the camera was monitored on the computer (E).

The ratios of protein-immobilised-silica to MA (*w/w*) used were 1:0.5 and 1:1. For coating, the required weights of protein-immobilised-silica and MA at desired ratios were introduced into the preheated (43 °C) high-pressure vessel. The vessel was then closed and liquid CO_2_ was pumped at a rate of 10 g/min until the pressure of 100 bar was achieved. The system was then left to equilibrate under stirring at 250 rpm for 10 min. The mixture in the vessel was stirred for another 15 min and then depressurised at a rate of 8 bar/minute to collect coated particles. The particles were collected, gently homogenised, sieved through a 100 μm sieve and stored at 5 °C until required for analysis and drug release.

### 3.4. Coating of Silica Particles by Solvent Evaporation

The protein immobilised S_FP_ particles were also coated by SE method as described by Pettit et al. to compare the efficacy of SCF coating [36]. The required amount of MA was dissolved in 15 mL of pentane and the bHb immobilised particles were suspended in it. The suspension was left to stir at 250 rpm in a fume hood at room temperature until the solvent was completely evaporated. Similarly to above, particles were collected, gently homogenised, sieved through a 100 µm sieve and stored at 5 °C until required for analysis and drug release. 

### 3.5. Analysis 

#### 3.5.1. bHb Quantification

A UV-Vis (UV-2550, Shimadzu, Kyoto, Japan) analysis was used for the quantification of bHb using a matched pair of quartz cuvette with a path length of 1 cm. The samples were always filtered through 0.45 μm filters before obtaining the spectrum between 200 to 700 nm. The concentrations were calculated using a calibration curve of bHb at 285 nm. 

#### 3.5.2. Conformational Integrity Determination

The conformational integrity of bHb after release from SCDDS was determined using circular dichroism (Chirascan qCD; Applied Photophysics, Leatherhead, U.K.). The CD spectrum was recorded using 1 mm pathlength cylindrical quartz cuvette in the far UV region between 190 to 260 nm with a bandwidth of 1 nm and sampling time of 2 s per point. The CD spectra obtained were then deconvoluted using CDNN software supplied by Applied Photophysics to obtain the fractional content of the five secondary structures. 

#### 3.5.3. Nitrogen (N_2_) Sorption Analysis

A BET analysis was performed using Gemini 2380 (Micromeritics instrument corporation, Hexton, U.K.) on S_FP_ and bHb immobilised S_FP_ particles. An amount of 10–15 mg of particles were weighed in the sample tube and degassed with nitrogen in order to remove any moisture or adsorbed gases. The degas conditions used for pure and protein adsorbed silica were 100 °C for 4 h and 40 °C for 8 h, respectively. Sample and reference tubes were then placed firmly inside the instrument and were dipped in liquid nitrogen. The air in the tubes was evacuated at a rate of 50–100 mmHg/min for 5 min. Complete BET adsorption and desorption isotherms were obtained at P/P0 from 0.05 to 1. Pore sizes were calculated using the BJH model.

#### 3.5.4. ATR-FTIR Analysis

The infrared spectra of S_FP_, bHb adsorbed S_FP_ and SCDDS particles were obtained using a Spectrum Two ATR-FTIR spectrometer (Perkin Elmer, Beaconsfield, UK). Approximately 1 to 2 mg of the particles was spread uniformly on the surface of a single reflection horizontal ATR accessory with a zinc selenide (ZnSe) crystal and adequate pressure was exerted on the sample using the pressure-arm. The spectra were collected from 4000–400 cm-1 range in transmission mode. Approximately 16 scans were collected for each spectrum with a spectrum resolution of 8 cm^−1^.

### 3.6. In-vitro Release Studies in Simulated Gastric (SGF) and Intestine (SIF) Fluids

*In-vitro* bHb release from SCDDS was performed in SGF and SIF without enzymes were prepared according to USP [49]. The media also contained 0.1% *w/v* (1 mg/mL) pluronic F-127 as a displacer to ensure that optimal release could be obtained from these formulations. SGF was prepared using 0.1 M HCl (pH 1.2) and SIF was obtained by mixing 25 mL of 0.2 M monobasic potassium phosphate with 11.2 mL of 0.2 M sodium hydroxide solutions, made up to 100 mL with de-ionised water. An amount of 100 mg of SCDDS was suspended in 100 mL of the dissolution media and continually stirred at 100 rpm during the release studies. The readings were recorded after 15, 60 and 120 min in SGF before switching to SIF to obtain the release at 135, 180, 240, 360 and 1440 min. An amount of 5 mL of sample was withdrawn at the abovementioned intervals and filtered using 0.45 μm syringe filter before determining the UV absorbance at 285 nm. The volume of the release media was kept constant by the addition of 5 mL of the fresh media after every sample withdrawal. The experiments were performed at 37.4 ± 0.2 °C and in triplicate. 

## 4. Conclusions

SCDDS for the oral delivery of biomolecules was prepared by the bHb immobilisation on mesoporous silica followed by coating of myristic acid via scCO_2_-assisted melt coating and conventional solvent evaporation methods. The advantage of using scCO_2_ processing was to obtain the coating at a lower temperature than the melting point of MA at atmospheric pressure and to avoid the use of an organic solvent. The *in-vitro* bHb release from myristic acid coated S_FP_ based SCDDS in intestinal fluid resulted in 45%–70% protein release depending on the myristic acid ratio and coating method. CD analysis on the protein released from SCDDS did not show any changes in the secondary structures, which proves that SCF processing at studied conditions had no effect on the conformation of bHb. This study showed that mesoporous silica and myristic acid can be used as core and shell materials to develop SCDDS for large therapeutic molecules to obtain pH-dependent release without causing any protein denaturation.

## Figures and Tables

**Table 1:** F1 and F2 calculations for the release of bHb from uncoated and SCDDS particles.

Time (min)	% Mean bHb Released ± S.D				
	*Control*	*S_FP_:MA_0.5_-SCF*	*S_FP_:MA_1.0_-SCF*	*S_FP_:MA_0.5_-PEN*	*S_FP_:MA_1.0_-SCF*
135	74.3 ± 1.1	48.2 ± 3.7	31.6 ± 2.5	55.2 ± 4.2	45.3 ± 5.0
180	85.4 ± 1.2	53.3 ± 2.6	42.2 ± 2.0	65.4 ± 3.8	52.8 ± 3.5
240	89.2 ± 1.0	59.7 ± 3.2	45.2 ± 1.5	71.3 ± 4.3	53.9 ± 4.1
360	89.2 ± 2.0	59.2 ± 4.1	46.2 ± 2.8	71.9 ± 4.8	55.5 ± 4.7
F1		35	51	22	39
F2		27	18	36	24

**Table 2:** Percentage content of secondary structures [mean ± SE (*n* = 2)] of untreated bHb and bHb released from MA-coated S_FP_ formulations.

Secondary Structure	bHb (Untreated)	bHb Released from S_FP_ Formulation	
		SCF Coated	Pentane Coated
**Helix**	60.5 ± 1.5%	59.1 ± 0.4%	60.7 ± 0.9%
Antiparallel	0.7 ± 0.1%	0.8 ± 0.0%	0.7 ± 0.1%
Parallel	4.9 ± 0.2%	5.1 ± 0.1%	4.9 ± 0.2%
Beta turns	12.4 ± 0.2%	12.5 ± 0.1%	12.3 ± 0.1%
Random coil	20.7 ± 1.0%	21.2 ± 0.1%	20.6 ± 0.7%
